# Epilepsy: A Multifaced Spectrum Disorder

**DOI:** 10.3390/bs13020097

**Published:** 2023-01-23

**Authors:** Luigi Vetri, Michele Roccella, Lucia Parisi, Daniela Smirni, Carola Costanza, Marco Carotenuto, Maurizio Elia

**Affiliations:** 1 Oasi Research Institute-IRCCS, Via Conte Ruggero 73, 94018 Troina, Italy; 2 Department of Psychology, Educational Science and Human Movement, University of Palermo, 90128 Palermo, Italy; 3 Department of Sciences for Health Promotion and Mother and Child Care “G. D’Alessandro”, University of Palermo, 90128 Palermo, Italy; 4 Clinic of Child and Adolescent Neuropsychiatry, Department of Mental Health, Physical and Preventive Medicine, University of Campania “Luigi Vanvitelli”, 81100 Caserta, Italy

Epilepsy is one of the most widespread chronic conditions, affecting about 50 million people worldwide. It consists of a group of neurological disorders characterized by recurrent epileptic seizures due to abnormal electrical activity in the brain. Epilepsy has numerous etiologies, some of which are still completely unknown; seizures can produce a plethora of different clinical manifestations, with a wide range of severities and varying impacts on individuals and their families.

In addition to seizures, the complex phenotypic picture of people with epilepsy is often complicated by the presence of psychiatric and medical comorbidities. Several diseases and conditions, including depression, anxiety, dementia, migraines, heart disease, peptic ulcers and arthritis, are more common in people with epilepsy than in the general population. Many patients also report cognitive problems. Therefore, epilepsy has been progressively recognized as a condition reaching well beyond seizures, and today it can be properly considered as a multifaced spectrum disorder.

The ILAE recently defined epilepsy as a brain disorder characterized not only by recurrent seizures, but also by psychosocial consequences [1].

On the other hand, the new classification (2017) recognizes comorbidities as a significant and essential element of the diagnostic process [2]. Similarly, the WHO, in a global report on epilepsy, also underlined the importance of coexisting psychiatric disorders and the need to recognize and treat them to ensure the adequate and effective global care of patients with epilepsy [3].

The neuropsychological definition in the clinical context is very important for understanding the different aspects of the disease—the clinical symptoms, psychiatric comorbidities, pharmacotherapy, social functioning, and the definition of cognitive impairments—and, therefore, this discipline is important for obtaining a correct diagnosis and planning appropriate neurorehabilitation interventions, considered as both cognitive recovery and compensation of deficits [4].

The neuropsychological evaluation in patients with epilepsy does not differ much from the evaluations conducted on patients with other neurological conditions or with other chronic diseases. Generally, the evaluation includes the main cognitive domains: intellectual and adaptive development, attention, memory and learning, language, visuo-spatial abilities, executive functions, sensorimotor skills, teaching skills, emotional behavioral functioning and quality of life [5].

Understanding the complex dynamics of interaction between clinical, cognitive, affective, psychosocial and neurobiological factors is very important for clinical practice in epileptology. In this case, the neuropsychologist is called to carry out the integration of the factors mentioned above, thereby contributing in a deeper way to the understanding of the patient with epilepsy and also favoring patient management.

Through the understanding of cognitive and neurobehavioral perturbations, due to the presence of seizures and/or underlying injuries of the nervous tissue, and to the effect of antiepileptic drugs on cognition, it is possible to trace the neuropsychological profile of every single patient. Therefore, this profile constitutes the sum of effects of epilepsy, of the bimodal contribution of the pharmacological treatment, of the presence of psychiatric comorbidities, and of the impact of a chronic disease and its related cognitive-affective problems in everyday life on the patient’s cognitive performances.

There are several factors characterizing epilepsy that can affect individuals’ neuropsychological performances. Therefore, it should come as no surprise that these repercussions are so frequent. The main factors are the etiology of seizures (probably the most important variable), the site and extent of the lesion causing epilepsy, seizure frequency, age of onset, seizure type, antiepileptic drugs side effects, and the frequency of subclinical epileptic anomalies.

A holistic evaluation of the patient with epilepsy is particularly appropriate in the case of pre-operative diagnostic monitoring of patients with drug-resistant epilepsy candidates for surgical therapies. In this case, the neuropsychological evaluation, assessing the patient’s cognitive-behavioral profile, can contribute to the definition of the topographic location of seizure discharges and to the anatomic demarcation of epileptic circuits. Moreover, the detailed neuropsychological exam can contribute to the prognosis of the surgical outcome, both in terms of the therapeutic monitoring of seizures and of possible psychiatric comorbidities.

Psychosocial factors in patients with epilepsy probably represent the factors that best define the psychopathology, particularly if they are associated with other stressful events, lack of awareness, maladaptation to the condition of epilepsy, perception of support or neglect and social stigma.

Psychopathology in patients with epilepsy, according to a recent review, is more frequent than once was believed. Mood and anxiety disorders are the more frequent psychiatric comorbidities, with a prevalence of 35% and 25.6%, respectively. Psychotic disorders are rarer, with a frequency of 5.7%; the frequency of obsessive compulsive disorder is even lower, while substance abuse is present in 8% of cases. Suicide risk is, overall, greater, at 9%. However, several authors appropriately propose that these results suggest introducing screening measures for anxiety–depressive disorder as part of the training for epilepsy care [6,7].

The presence of psychiatric comorbidities does not only influence quality of life, but it also negatively affects the global prognosis of the individual with epilepsy. An interesting recent study showed how, compared to the general population, both subjects with both epilepsy and psychiatric comorbidities and those without comorbidities present a higher mortality risk. However, subjects with epilepsy and at least a psychiatric comorbidity have a morality rate 1.4 times higher compared with people who have epilepsy without a psychiatric illness, after adjusting for age and sex [8].

Psychiatric diseases are not the only ones complicating the clinical profile of subjects with epilepsy; endocrine/metabolic disorders, respiratory diseases, urogenital diseases and cardiovascular diseases are up to eight times more frequent and contribute to substantially increase the burden for people with epilepsy [9,10].

Moreover, during infancy and adolescence, people with epilepsy experience cognitive and behavioral problems, and these difficulties are frequently related to neurodevelopmental disorders such as autism spectrum disorder, attention-deficit hyperactivity disorder, and learning challenges. Epilepsy and neurodevelopmental disorders co-occur, according to the overlap model in which these conditions shared a common genetic susceptibility [11].

A holistic view of patients with epilepsy has many potential implications for the condition’s management and treatment. Unfortunately, the scientific evidence about the effect of antiepileptic drugs on comorbid psychiatric disorders remains suboptimal, and more often in clinical practice a psychotherapeutic approach should be taken into consideration to avoid polypharmacotherapy.

In conclusion, for a better global care of the patient with epilepsy, a new paradigm is mandatory, encompassing the broader range of cognitive and behavioral comorbidities of epilepsy, reflecting the heterogeneity of their phenotype, and enhancing the complexity of etiological factors such as neurobiological diversity, genomics influences, and resilience factors. Ultimately, this will place the focus on the individual patient and raise the possibility of a precision medicine approach [12].
